# Supplementary material for: Effectiveness and Safety of Ayurvedic Medicines in Type 2 Diabetes Mellitus Management: A Systematic Review and Meta-Analysis
Source: Front Pharmacol. 2022 Jun 8;13:821810. doi: 10.3389/fphar.2022.821810 (PMC9213670; doi:10.3389/fphar.2022.821810)
Supplement: Supplementary file 1 [file DataSheet1.zip › Supplementary Material/Forest and Funnel Plots/Trigonella foenum-graecum/LDL-C.pdf]

Trigonella foenum-graecum - LDL-C

Study	Intervention N	Intervention Mean	Intervention SD	Comparator N	Comparator Mean	Comparator SD	Mean Diff. with 95% CI	Weight (%)
Gupta 2001	12	111.00	37.00	13	138.70	28.00	-27.70 [-53.29, -2.11]	10.22
Rafraf 2014	44	52.86	20.38	44	45.73	22.93	7.13 [-1.93, 16.19]	19.67
Singh 2016	20	100.03	20.18	20	111.97	28.49	-11.94 [-27.24, 3.36]	15.80
Gholaman 2018	10	190.70	15.36	10	201.50	14.40	-10.80 [-23.85, 2.25]	17.22
Kandhare 2018	48	121.98	31.30	53	122.51	32.40	-0.53 [-12.98, 11.92]	17.60
Rashid 2019	32	73.08	18.56	32	98.21	19.72	-25.13 [-34.51, -15.75]	19.49
**Overall**							-10.17 [-21.21, 0.88]	

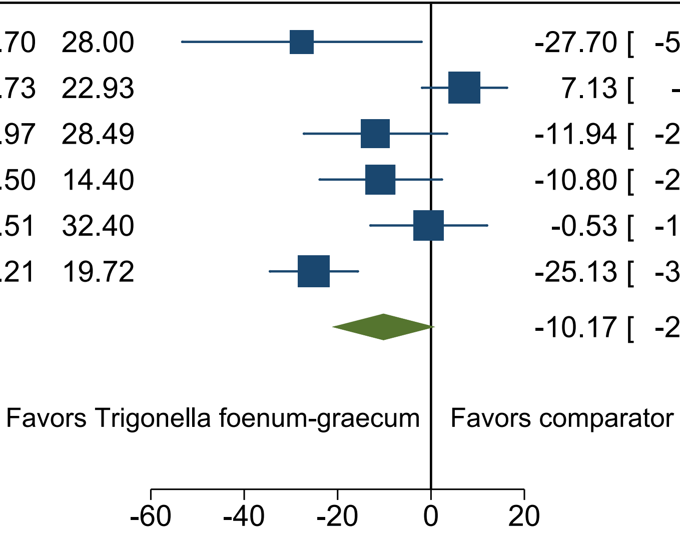

Heterogeneity: $\tau^2 = 139.97$, $I^2 = 77.85\%$, $H^2 = 4.51$

Test of $\theta_i = \theta_j$: Q(5) = 27.54, p = 0.00

Test of $\theta = 0$: z = -1.80, p = 0.07

Random-effects REML model
